# Predicting Survival in Patients with Pancreatic Cancer by Integrating Bone Marrow FDG Uptake and Radiomic Features of Primary Tumor in PET/CT

**DOI:** 10.3390/cancers13143563

**Published:** 2021-07-16

**Authors:** Jeong Won Lee, Sang-Heum Park, Hyein Ahn, Sang Mi Lee, Su Jin Jang

**Affiliations:** 1 Department of Nuclear Medicine, Catholic Kwandong University College of Medicine, International St. Mary’s Hospital, Incheon 22711, Korea; sads00@naver.com; 2 Division of Gastroenterology and Hepatology, Department of Internal Medicine, Soonchunhyang University Cheonan Hospital, Cheonan 31151, Korea; pparksh@schmc.ac.kr; 3 Department of Pathology, Soonchunhyang University Cheonan Hospital, Cheonan 31151, Korea; hyein.ahn@schmc.ac.kr; 4 Department of Nuclear Medicine, Soonchunhyang University Cheonan Hospital, Cheonan 31151, Korea; 5 Department of Nuclear Medicine, CHA Bundang Medical Center, CHA University, Seongnam-si 13496, Korea

**Keywords:** bone marrow, FDG PET/CT, pancreatic cancer, prognosis, texture analysis

## Abstract

**Simple Summary:**

FDG uptake of bone marrow (BM) is known to reflect the degree of host inflammatory response to cancer cells and showed significant association with survival in diverse kinds of cancers. The aim of this retrospective study was to evaluate the prognostic significance of FDG uptake of BM and to investigate whether integrating FDG uptake of BM and radiomic features of primary tumors could improve the prediction of overall survival (OS) in patients with pancreatic cancer. In multivariable survival analysis, along with total lesion glycolysis (TLG) and first-order entropy of primary tumor lesions, FDG uptake of BM was an independent predictor of OS. We designed a PET/CT scoring system based on the cumulative scores of tumor factors (TLG and first-order entropy) and host factors (FDG uptake of BM). This scoring system was able to stratify the patients with three distinct prognostic groups independent of clinical stage and treatment modality.

**Abstract:**

The purpose of this study was to evaluate the prognostic significance of FDG uptake of bone marrow (BM SUV) and to investigate its role combined with radiomic features of primary tumors in improving the prediction of overall survival (OS) in patients with pancreatic cancer. We retrospectively enrolled 65 pancreatic cancer patients with staging FDG PET/CT. BM SUV and conventional imaging parameters of primary tumors including total lesion glycolysis (TLG) were measured. First-order and higher-order textural features of primary cancer were extracted using PET textural analysis. Associations of PET/CT parameters of bone marrow (BM) and primary cancer with OS were assessed. BM SUV as well as TLG and first-order entropy of pancreatic cancer were significant independent predictors of OS in multivariable analysis. A PET/CT scoring system based on the cumulative scores of these three independent predictors enabled patient stratification into three distinct prognostic groups. The scoring system yielded a good prognostic stratification based on subgroup analysis irrespective of tumor stage and treatment modality. BM SUV was an independent predictor of OS in pancreatic cancer patients. The PET/CT scoring system that integrated PET/CT parameters of primary tumors and BM can provide prognostic information in pancreatic cancer independent of tumor stage and treatment.

## 1. Introduction

Pancreatic cancer is notable for its poor prognosis with a 5-year overall survival (OS) rate of only 9% [1]. Recent clinical trials with curative radical resection followed by adjuvant treatment showed significant improvement in OS; however, only 20% of newly diagnosed pancreatic cancers are potentially resectable [2,3]. Furthermore, even if patients received curative surgical resection, recurrence occurred in up to 72–79% of the patients with 5-year OS rates of only 20–38% [2,4,5]. Therefore, a number of studies have been performed to investigate the prognostic factors for stratification of clinical outcomes in patients with pancreatic cancer beyond the TNM staging system [6]. In addition to prognostic factors originating from tumor intrinsic properties such as lymph node metastasis, perineural invasion, and serum carbohydrate antigen 19-9 (CA19-9) levels, several recent studies have focused on the role of host inflammatory response in the progression of pancreatic cancer [4,7,8,9]. Among host immune cells, neutrophil and myeloid-derived suppressor cells promote growth and migration of pancreatic cancer cells, and increased serum inflammatory markers such as neutrophil-to-lymphocyte ratio (NLR) and platelet-to-lymphocyte ratio (PLR) showed significant association with worse clinical outcomes in patients with pancreatic cancer [7,8,9,10].

F-18 fluorodeoxyglucose (FDG) positron emission tomography/computed tomography (PET/CT) is a diagnostic imaging modality with significant clinical value in detecting metastatic lesions and predicting clinical outcomes of patients with pancreatic cancer [11,12]. In studies evaluating the prognostic value of FDG PET/CT, conventional PET/CT parameters of pancreatic cancer such as maximum standardized uptake value (SUV), metabolic tumor volume (MTV), and total lesion glycolysis (TLG) have been generally used to estimate tumor metabolism [13]. Additionally, over the last decade, several studies have investigated radiomic features of tumors extracted from texture analysis of PET/CT images to quantify intratumoral metabolic heterogeneity and showed significant association of PET/CT imaging features with survival in patients with pancreatic cancer, suggesting the role of radiomic features as prognostic imaging biomarkers [6,14]. Meanwhile, in contrast to studies that focused on imaging features of cancer lesions, other recent studies evaluated the degree of host inflammatory response to cancer using FDG PET/CT [15,16]. FDG uptake of bone marrow (BM) is known to be associated with the production of myeloid cells, including neutrophils and macrophages, and showed a significant correlation with the degree of the systemic inflammatory response of the host [15,16,17,18]. Furthermore, a number of studies have demonstrated the significant positive association between FDG uptake of BM and survival in diverse kinds of cancers [15,16,17,19]. However, until now, no studies have reported the prognostic significance of BM FDG uptake in patients with pancreatic cancer.

Hence, in the present study, we aimed to evaluate the prognostic value of FDG uptake of BM for predicting OS in patients with pancreatic cancer. Further, based on the “seed and soil theory” [16,20], we also investigated whether integrating FDG uptake of BM and radiomic features of pancreatic cancer could further improve prognostic stratification.

## 2. Materials and Methods

### 2.1. Patients

We retrospectively reviewed the electronic medical records of 97 patients who were histopathologically diagnosed with pancreatic ductal adenocarcinoma in Soonchunhyang University Cheonan Hospital between January 2012 and December 2017. Of them, 65 patients who underwent FDG PET/CT for staging work-up, in addition to blood tests and contrast-enhanced abdominopelvic CT, and received curative or palliative treatment for pancreatic cancer were finally enrolled in the study. The patients (1) who had no staging FDG PET/CT scan (*n* = 6), (2) who received only supportive care without any treatment (*n* = 14), (3) who had a previous history of other malignant disease (*n* = 3), (4) who carried primary pancreatic tumors with low FDG uptake inadequate for accurate tumor delineation for radiomic analysis (*n* = 4), and (5) who had insufficient primary tumor volumes for radiomic analysis, specifically, tumor volumes <64 voxels (*n* = 5), were excluded from the study. Based on clinical stage and patient clinical condition, all the enrolled patients received either curative or palliative treatment and were regularly followed up with blood tests and imaging studies. OS was defined as the duration between the date of the initial treatment and the date of death or the last follow-up in our medical center.

### 2.2. FDG PET/CT

FDG PET/CT images were acquired using a dedicated PET/CT scanner (Biograph mCT 128 scanner, Siemens Healthineers, Knoxville, TN, USA). All patients were instructed to fast for at least 6 h before the PET/CT scan. Blood glucose levels were required to be less than 200 mg/dL at the time of FDG injection. Approximately 60 min after the intravenous administration of FDG (4.07 MBq/kg), PET/CT was performed from the base of the skull to the proximal third of the thigh. Initially, a non-contrast-enhanced CT was performed at 100 mA and 120 kVp, and, thereafter, a PET scan was performed for 1.5 min in each bed position under the three-dimensional acquisition mode. PET images were reconstructed with a point spread function based Gauss and Allpass filter algorithm and time-of-flight reconstruction (two iterations and 21 subsets) with a 128 × 128 matrix using CT images for attenuation correction.

### 2.3. PET/CT Image Analysis

**Figure 1:**
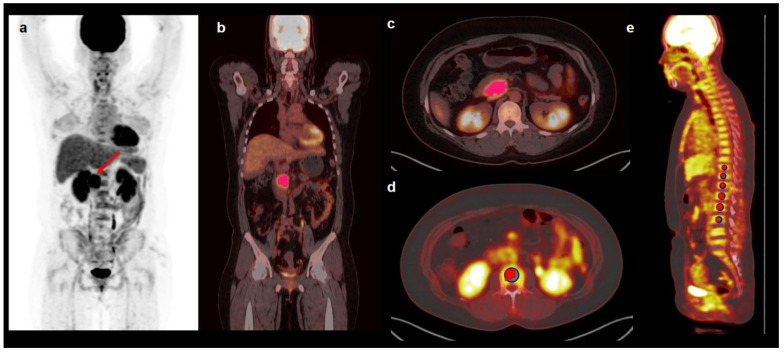
Maximal intensity projection image (**a**), coronary image (**b**), transaxial images (**c**,**d**), and sagittal image (**e**) of FDG PET/CT showing an example of VOIs for measuring imaging features of pancreatic cancer and FDG uptake of BM. A 43-year-old woman underwent FDG PET/CT for staging work-up of pancreatic cancer with a maximum SUV of 9.55 (arrow on **a**). To delineate pancreatic cancer lesion, a VOI was manually drawn around the primary tumor, and an area that showed SUV higher than the threshold value of 4.22 determined by Nestle’s adaptive threshold method was selected within the VOI (pink color in **b**,**c**). To measure FDG uptake of BM, six spheroidal VOIs were drawn over the vertebral body of the thoracic and lumbar spine, and an isocontour using a cutoff SUV of 75% of the maximum SUV was automatically generated within each VOI (red color in **d** and **e**). Mean SUV of voxels within the isocontour was measured and defined as BM SUV.

For the extraction of textural features of primary tumors on PET images, the open-source LIFEx software version 7.0.0 (www.lifexsoft.org, accessed on 16 May 2021) was used [21]. For tumor segmentation, a volume of interest (VOI) was manually drawn around the primary tumor lesion, and, subsequently, the delineation of the primary tumor within VOI was automatically performed with Nestle’s adaptive threshold method as follows: tumor threshold = 0.3 × (mean standardized uptake value (SUV) of voxels with an uptake greater than 70% of maximum SUV of tumor lesion) + (mean SUV of background voxels) (Figure 1) [22,23,24]. The margins of all primary tumor lesions were manually inspected by experienced nuclear medicine physicians to avoid FDG uptake of adjacent organs included in the VOIs. Before calculating textural parameters from VOIs of primary tumors, the intensity levels of FDG uptake were resampled into 64 relative levels between zero and maximum values of FDG uptake [25]. For each patient, a total of 41 textural parameters were extracted from VOI of primary tumors on PET images: 4 conventional imaging parameters, 6 first-order textural features, and 31 higher-order textural features. Four conventional imaging parameters comprised maximum SUV, peak SUV, MTV, and TLG. Six first-order textural features consisted of four SUV histogram-based features (skewness, kurtosis, entropy, and energy) and two shape features (sphericity and compacity). Higher-order textural features included 6 grey-level co-occurrence matrix (GLCM) features, 3 neighborhood grey-level different matrix features, 11 grey-level run-length matrix features, and 11 grey-level zone-length matrix (GLZLM) features (Table S1).

For estimating FDG uptake of BM, Osirix MD 10.0 software (Pixmeo, Geneva, Switzerland) was used. According to methods used in previous studies, two PET/CT imaging parameters of BM, mean FDG uptake of BM (BM SUV) and bone marrow-to-liver uptake ratio (BLR), were measured [15,16,19]. Spheroid-shaped VOIs were manually drawn over the vertebral body of each of six vertebrae in the thoracic and lumbar spine (Figure 1). We excluded vertebrae with severe osteoarthritic change, benign bone tumor, compression fracture, or postoperative change of previous spinal surgery from the measurement. An isocontour using a cutoff SUV of 75% of the maximum SUV was generated within each VOI, and the mean SUV of voxels within isocontour was defined as SUV of the vertebral body. This method of using the cutoff SUV of 75% of the maximum SUV showed substantial agreement between observers in a previous study [15]. The average SUV of six vertebral bodies was defined as BM SUV. For measuring FDG uptake of the normal liver, a 2 cm sized spheroidal-shaped VOI was drawn in the right lobe of the liver at a location without metastatic lesion on imaging studies and the mean SUV of VOI was measured. BLR was calculated for each patient using BM SUV and the mean SUV of the liver.

### 2.4. Statistical Analysis

For each patient, NLR and PLR were calculated using the blood tests performed during the staging work-up. After evaluating the normality of distribution using the Shapiro–Wilk test, Spearman rank correlation coefficients were calculated to evaluate the relationships of BM SUV and BLR with NLR and PLR. To evaluate the association of FDG PET/CT parameters and clinical factors with OS, a Cox proportional hazards regression model was used for univariable and multivariable analyses, and the Harrell’s concordance index (C-index) was estimated. The continuous variables included in the survival analysis were dichotomized according to the optimal cutoff values determined by maximally selected chi-square test. For PET/CT imaging parameters that showed statistical significance in univariable survival analysis, multivariable analysis was performed after adjusting for age and sex. Considering the rule of thumb for the Cox regression model [26], only one parameter that had the highest value of C-index was selected for each BM and conventional imaging parameter in pancreatic cancer. For first-order and higher-order textural features of pancreatic cancer, imaging parameters that showed significant correlation with each other were assessed in a separate model; therefore, two different models were reconstructed for multivariable analysis. Using the PET/CT parameters that remained significant predictors for OS in multivariable analysis, we devised a PET/CT scoring system for predicting OS. The independent predictors in multivariable analysis were categorized into tumor factors (imaging features of pancreatic cancer) and host factors (BM imaging parameters). A score was assigned for each factor (score 1 for presence of each tumor factor and score 2 for presence of host factor). A score of 0 was assigned for the absence of tumor or host factor. The scoring system was based on the summation of scores of tumor and host factors, reflecting the number of positive tumor and host factors identified in each patient, and the prognostic value of the scoring system for predicting OS was assessed. The Kaplan–Meier method was used to estimate OS curves of the variables. Statistical analyses were performed using MedCalc Statistical Software version 20 (MedCalc Software Ltd., Ostend, Belgium) and R software version 4.0.5 (The R Foundation for Statistical Computing, Vienna, Austria). *p*-values < 0.05 were regarded as statistically significant.

## 3. Results

### 3.1. Patient Characteristics

The clinical characteristics of the 65 enrolled patients with pancreatic cancer are presented in Table 1. Staging work-up examinations revealed regional lymph node metastasis in 30 patients (46.2%) and distant metastasis in 12 patients (18.5%). Of the patients, 37 (56.9%) underwent surgical resection for pancreatic cancer lesions, and among them, 25 patients received adjuvant treatment after the surgery. At the time of analysis, 37 patients (56.9%) died during the follow-up. The median OS of the patients was 14.1 months (range, 2.0–61.0 months) with a 1-year OS rate of 62.7%. 

On FDG PET/CT, 12 patients (18.5%) showed BM SUV higher than the mean SUV of the liver (BLR > 1.0). The correlation between BM imaging parameters and serum inflammatory markers was significant, whereas a weak correlation existed between BM SUV and NLR (*p* = 0.027; correlation coefficient, 0.274) and between BLR and PLR (*p* = 0.049; correlation coefficient, 0.245).

### 3.2. Survival Analysis

The prognostic significance of FDG PET/CT parameters of BM and primary cancer for predicting OS was assessed along with clinical factors. All continuous variables were dichotomized by the specific cutoff values determined by maximally selected chi-square test. In univariable survival analysis, both BM SUV and BLR showed significant association with OS, showing worse OS in patients with increased FDG uptake of BM (*p* < 0.05; Table 2). Among PET/CT textural features of pancreatic cancer, peak SUV, MTV, TLG, first-order entropy, GLCM energy, GLCM entropy, and GLZLM zone length nonuniformity were significant predictors for OS (*p* < 0.05; Table 2 and Table S2). Among clinical factors, age, clinical TNM stage, serum carcinoembryonic antigen (CEA), serum CA19-9, NLR, and treatment modality showed significant association with OS (*p* < 0.05; Table 2).

**Figure 2:**
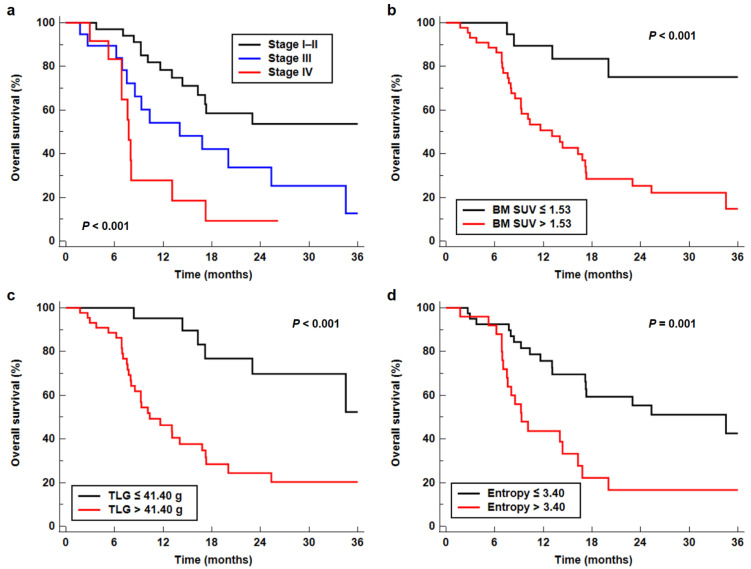
Kaplan–Meier curves for OS according to clinical TNM stage (**a**), BM SUV (**b**), TLG (**c**), and first-order entropy (**d**).

Among the PET/CT parameters, those that showed statistical significance in the univariable analysis were included in multivariable survival analysis along with NLR and clinical TNM stage. Considering the numbers of PET/CT parameters as compared with the number of events in the enrolled patients, only BM SUV, which has a higher C-index than BLR, and TLG, which has the highest value of C-index among peak SUV, MTV, and TLG, were included in the multivariable analysis representing BM and conventional imaging parameters, respectively. First-order entropy and GLCM entropy showed a significantly strong positive correlation with each other (*p* < 0.001; correlation coefficient, 0.972); hence, only first-order entropy was selected. Because first-order entropy also showed significant correlation with GLCM energy (*p* < 0.001, correlation coefficient, −0.544) and GLZLM zone length nonuniformity (*p* < 0.001; correlation coefficient, 0.508), two different multivariable models were developed to evaluate prognostic significance. After adjusting for age and sex, the results of the multivariable analysis demonstrated that clinical TNM stage, BM SUV, TLG, and first-order entropy were independent prognostic factors for predicting OS (*p* < 0.05; Table 3). Meanwhile, NLR, GLCM energy, and GLZLM zone length nonuniformity failed to show statistical significance in multivariable analysis (*p* > 0.05). In Kaplan–Meier analysis, the median OSs of the patients with clinical stages I–II, III, and IV were 42.1 months (95% confidence interval (CI), 16.3–42.1 months), 14.0 months (95% CI, 7.5–25.3 months), and 7.7 months (95% CI, 5.2–13.1 months), respectively (*p* < 0.001; Figure 2a). Patients with low values of BM SUV (*p* < 0.001; 42.1 months vs. 13.1 months), TLG (*p* < 0.001; 42.1 months vs. 10.3 months), and first-order entropy (*p* = 0.001; 34.5 months vs. 9.3 months) had significantly higher median OS than those with high values (Figure 2b–d).

### 3.3. PET/CT Scoring System for Predicting Survival

**Figure 3:**
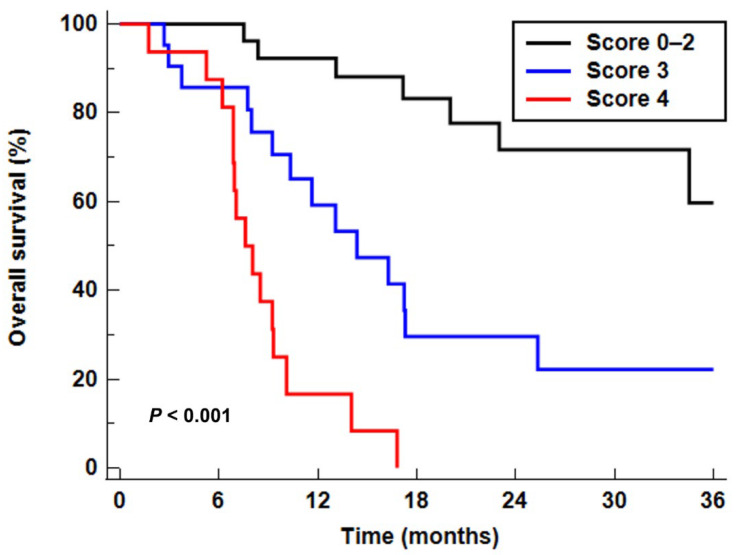
Kaplan–Meier curves for OS according to the PET/CT scoring system.

Using three independent predictors among PET/CT parameters (BM SUV, TLG, and first-order entropy), we designed a scoring system for predicting OS that integrated “seed” (tumor factors, TLG and first-order entropy) and “soil” (host factor, BM SUV) parameters. For tumor factors, those with high values (TLG > 41.40 or first-order entropy > 3.40) were assigned a score of 1 and those with low values (TLG ≤ 41.40 or first-order entropy ≤ 3.40) were assigned a score of 0. For the host factor, BM SUV > 1.53 and ≤ 1.53 were assigned scores of 2 and 0, respectively. Therefore, the summed scores ranged from 0 to 4. This scoring system allowed prognostic stratification of the patients into three groups with distinct OS (*p* < 0.001; Figure 3): the patient group with a score of 0–2 (patients with low values of all tumor and host factors or high values for either tumor or host factors), the patient group with a score of 3 (patients who had high values of host factor and one of the tumor factors), and the patient group with a score of 4 (patients who showed high values of host and both tumor factors). Compared with patients with a score of 0–2 (median OS, 42.1 months), patients with a score of 3 (*p* = 0.004; hazard ratio, 3.71; median OS, 14.4 months) or 4 (*p* < 0.001; hazard ratio, 14.52; median OS, 7.6 months) had significantly worse OS (Table 4). In the Harrell’s C statistical analysis, the scoring system showed great discriminative ability in predicting OS with a C-index of 0.793 (95% CI, 0.674–0.883) which was higher than that of clinical TNM stage (0.690; 95% CI, 0.617–0.794).

To further evaluate the ability of PET/CT scoring system in prognostic stratification according to clinical TNM stage and treatment, the study patients were classified into two subgroups based on clinical TNM stage (stage I–II vs. stage III–IV) and treatment (surgical resection vs. other treatments). Afterward, the prognostic significance of the PET/CT scoring system was assessed in those subgroups (Table 5). In all subgroup analyses, patients with scores of 3 and 4 showed significantly worse OS than those with scores 0–2 (*p* < 0.05), indicating the good prognostic value of the stratification system independent of tumor stage and treatment. The C-index of the combination of clinical TNM stage and PET/CT scoring system in predicting OS was 0.827 (95% CI, 0.713–0.910).

## 4. Discussion

In patients with malignant diseases, increased FDG uptake in BM is often encountered, showing higher FDG uptake of BM than normal liver tissue among 8.2–17.2% of them [15,19,27]. Because granulopoiesis rather than erythropoiesis strongly contributes to FDG uptake of BM and FDG uptake of BM shows a significant correlation with serum inflammatory markers, including serum C-reactive protein, neutrophil count, NLR, and PLR, the FDG uptake of BM is considered as an imaging parameter for evaluating the degree of systemic inflammatory response [15,17,19,28,29]. Furthermore, a number of studies have demonstrated a significant association between FDG uptake of BM and recurrence-free survival and OS in various malignant diseases including head and neck cancer, lung cancer, breast cancer, gastric cancer, colon cancer, and gynecologic cancer, consistently showing worse survival in patients with high BM FDG uptake [15,17,19,27,29,30,31,32,33]. In the present study, BM imaging parameters showed a significant positive association with serum inflammatory markers, and BM SUV was an independent predictor of OS in multivariable analysis after adjusting for age, sex, and TNM stage, indicating that BM SUV could have prognostic significance in patients with pancreatic cancer as well. 

The underlying mechanism responsible for the association between FDG uptake of BM and prognosis has yet to be elucidated, but a possible causative mechanism was suggested by a recent study [17]. Animal experiments in that study revealed that increased tumor-derived granulocyte colony-stimulating factor (G-CSF) levels significantly increased the FDG uptake of BM on PET/CT images and animals with increased levels of G-CSF displayed increased myeloid-derived suppressor cells in the blood and tumor tissue [17]. Myeloid-derived suppressor cells are immature myeloid cells with polymorphonuclear or mononuclear morphology [8]. These cells are known to suppress antitumoral immunity; induce a tumor-permissive microenvironment; and contribute to tumor growth, angiogenesis, and metastasis, resulting in worse clinical outcomes [8,34]. Therefore, immune suppression mediated by myeloid-derived suppressor cells might lead to poor prognosis of patients with increased FDG uptake of BM [17]. In addition, considering the significant positive correlation between the degree of FDG uptake and macrophage infiltration in the tumors, FDG uptake of BM could be affected by the metabolic activity of BM-derived macrophages [18]. BM-derived macrophages can convert to tumor-associated macrophages in the tumor microenvironment, which contributes to a favorable microenvironment for tumor growth [18,35]. In previous studies, increased myeloid-derived suppressor cells and tumor-associated macrophages were also significantly associated with poor response to treatment and worse survival in patients with pancreatic cancer [8,35,36,37]. Based on these results, it is plausible that FDG uptake of BM has a significant association with OS in patients with pancreatic cancer as shown in our study, suggesting that FDG uptake of BM might be used to estimate host immune conditions in pancreatic cancer. Recently, several clinical trials investigated the effects of therapeutic agents targeting myeloid-derived suppressor cells [8], and patients with high FDG uptake of BM might be good candidates for these agents. In addition to FDG uptake of BM, NLR, which is a well-known serum inflammatory marker, also showed a significant predictive value for OS in univariable analysis. However, in multivariable analysis, only BM SUV was an inflammatory response biomarker that showed statistical significance, which might suggest that BM SUV might be more useful for predicting OS than serum inflammatory markers.

Among textural features of pancreatic cancer, TLG and first-order entropy were independent predictors of OS in this study. TLG is a volumetric PET/CT parameter that reflects metabolically active tumor burden and has been already shown a significant association with survival in several studies with pancreatic cancer [6,12,13]. First-order entropy of PET/CT images measured via SUV-histogram analysis was found to be one of the textural features insensitive to PET image reconstruction methods and showed an excellent inter-rater agreement for the measurement [6,38,39]. It describes the randomness of SUV from the voxel value frequency distribution, and increased entropy is known to be associated with increased intratumoral metabolic heterogeneity [6]. Similar to the results of our study, a previous study of 137 patients diagnosed with pancreatic ductal adenocarcinoma demonstrated that first-order entropy was independently associated with survival, showing improved survival in patients with low-entropy cancer lesions [6]. Based on the results of the present study, both tumor metabolic burden and intratumoral metabolic heterogeneity are significant independent tumor factors for predicting OS in patients with pancreatic cancer.

In 1889, the so-called “seed and soil theory” was proposed, which stated that the spread of cancer cells is driven by the interaction between cancer cells and host cells [20,40]. Since then, growing evidence has suggested that the host microenvironment plays an essential role in the growth and metastasis of cancer cells, as well as the biological characteristics of cancer cells [20,40]. Because immune cells derived from the BM such as myeloid-derived suppressor cells and macrophages significantly influence the host organ microenvironment, the degree of BM FDG uptake, which reflects the degree of production and metabolic activity of BM-derived immune cells, can be used to estimate the immune microenvironment of the host organ [17,18,35,41]. Hence, a prognostic model that combines tumor factors and BM imaging parameters that reflect host factors could improve the prediction of the prognosis of cancer patients when compared with a model only with tumor intrinsic factors. In previous studies of patients with breast cancer and non-small-cell lung cancer, a prognostic model that combined the imaging features of both primary tumor and BM predicted clinical outcomes more accurately than tumor parameters alone [16,42]. Similarly, because independent prognostic PET/CT parameters in our study comprised both tumor (TLG and first-order entropy) and host factors (BM SUV), we devised a PET/CT scoring system for predicting OS that integrated tumor features and host condition. Using this scoring system, we successfully stratified the prognosis of patients with pancreatic cancer. The results of our study demonstrated that patients carrying high values of both tumor and host factors in FDG PET/CT showed significantly worse OS than those who had high values of only one of these or had low values of both factors. Patient subgroups with scores of 0–2, 3, and 4 showed similar median survival compared to those at clinical stages I–II, III, and IV, respectively. Furthermore, this scoring system carried a significant value for prognostic stratification in both patient subgroups with early and advanced stages and in both patient subgroups with surgical resection and other treatments, suggesting that the integration of imaging parameters of tumor and host could provide further prognostic stratification beyond TNM stage. The results of this study provide imaging evidence that supports “seed and soil theory” in pancreatic cancer. Further aggressive treatment and surveillance strategies would be needed for patients with high scores in the scoring system representing robust seed and permissive soil. 

The present study shows several limitations. First, this study was a retrospective review of patients who had undergone various treatment modalities in a single medical center; hence, there might have been an inherent risk of selection bias. Second, because of the limited number of enrolled patients, we were unable to perform cross-validation of the PET/CT scoring system. Further external validation of our results in a larger patient cohort is necessary. Third, the calculation of textural features on PET/CT images is known to be influenced by various factors such as the method of tumor segmentation and partial volume effect, which might affect the study results [6]. Fourth, because of the small number of patients in the subgroup analysis, the results of the subgroup analysis may not be generalizable. Finally, due to the retrospective nature of the study, we could not evaluate other factors that represent the host immune condition such as interleukin 6, transforming growth factor-beta, and BM biopsy results. Further studies based on histopathological and laboratory analyses are needed to delineate the underlying mechanism of the association between FDG uptake of BM and prognosis in patients with pancreatic cancer.

## 5. Conclusions

In this study, BM SUV, as well as TLG and first-order entropy of primary tumor lesion, was independently associated with OS after adjustment for age, sex, NLR, and clinical TNM stage in patients with pancreatic cancer. Patients with high values of BM SUV, TLG, and first-order entropy showed significantly worse OS than those with low values. Using these three independent prognostic factors in FDG PET/CT, we designed a scoring system that integrated both tumor and host imaging parameters. This scoring system was able to stratify the patients into three distinct prognostic groups irrespective of tumor stage and treatment modality. Integrating imaging parameters of BM and primary cancer on FDG PET/CT can facilitate the prediction of prognosis in patients with pancreatic cancer.

## Figures and Tables

**Table 1:** Clinical characteristics of patients with pancreatic cancer (*n* = 65).

Characteristics		Number of Patients (%)
Age (years)		66 (40–89) *
Sex	Men	34 (52.3%)
	Women	31 (47.7%)
Tumor location	Head/neck	37 (56.9%)
	Body	13 (20.0%)
	Tail	15 (23.1%)
Tumor size (cm)		3.7 (1.9–8.7) *
T classification	T1–T2	9 (13.8%)
	T3–T4	56 (86.1%)
N classification	N0	35 (53.8%)
	N1	30 (46.2%)
M classification	M0	53 (81.5%)
	M1	12 (18.5%)
Clinical TNM stage	I	6 (9.2%)
	II	28 (43.1%)
	III	19 (29.2%)
	IV	12 (18.5%)
Serum CEA (ng/mL)		4.32 (0.56–100,000.00) *
Serum CA19-9 (U/mL)		138.5 (0.6–4628.0) *
NLR		2.26 (0.80–21.74) *
PLR		150.22 (56.96–1574.51) *
PET/CT parameters	Maximum SUV of primary tumor	7.01 (3.55–22.47) *
	MTV of primary tumor (cm^3^)	15.58 (1.91–77.37) *
	TLG of primary tumor (g)	55.54 (10.11–845.87) *
	BM SUV	1.74 (1.13–2.91) *
	BLR	0.86 (0.49–1.86) *
Treatment	Surgical resection	37 (56.9%)
	Concurrent chemotherapy	13 (20.0%)
	Chemotherapy alone	11 (16.9%)
	Radiotherapy alone	4 (6.2%)

* Median value (range); BM, bone marrow; BLR, bone marrow-to-liver uptake ratio; CA19-9, carbohydrate antigen 19-9; CEA, carcinoembryonic antigen; MTV, metabolic tumor volume; NLR, neutrophil-to-lymphocyte ratio; PET/CT, positron emission tomography/computed tomography; PLR, platelet-to-lymphocyte ratio; SUV, standardized uptake value; TLG, total lesion glycolysis.

**Table 2:** Univariable analysis of PET/CT imaging parameters showing statistical significance and clinical factors for predicting overall survival.

Variables		*p*-Value	Hazard Ratio (95% Confidence Interval)	C-Index
Age (≤65 years vs. >65 years)		0.020	2.26 (1.14–4.48)	0.632
Sex (women vs. men)		0.346	0.73 (0.38–1.41)	0.574
Clinical TNM stage	Stage I–II vs. stage III	0.025	2.41 (1.12–5.20)	
	Stage I–II vs. stage IV	<0.001	5.07 (2.18–11.78)	0.690
Serum CEA (≤5.00 ng/mL vs. >5.00 ng/mL)		0.002	2.93 (1.49–5.74)	0.663
CA19-9 (≤103.0 U/mL vs. >103.0 U/mL)		0.021	2.28 (1.13–4.59)	0.632
NLR (≤4.17 vs. >4.17)		0.047	2.03 (1.01–4.10)	0.642
PLR (≤217.54 vs. >217.54)		0.068	1.85 (0.96–3.57)	0.625
Treatment (surgery vs. others treatments)		0.048	1.91 (1.02–3.70)	0.602
BM imaging parameters	BM SUV (≤1.53 vs. >1.53)	0.002	4.47 (1.73–11.51)	0.678
	BLR (≤0.79 vs. >0.79)	0.003	4.76 (1.68–13.54)	0.658
Conventional PET/CT parameters	Peak SUV (≤6.65 vs. >6.65)	0.048	2.90 (1.01–6.53)	0.622
	MTV (≤15.60 cm^3^ vs. >15.60 cm^3^)	0.021	2.18 (1.12–4.21)	0.619
	TLG (≤41.40 g vs. >41.40 g)	0.002	3.80 (1.65–8.72)	0.655
First-order textural parameter	Entropy (≤3.40 vs. >3.40)	0.002	2.87 (1.47–5.61)	0.650
Higher-order textural parameters	GLCM energy (≤0.012 vs. >0.012)	0.011	0.43 (0.22–0.82)	0.648
	GLCM entropy (≤6.45 vs. >6.45)	0.027	2.10 (1.09–4.05)	0.640
	GLZLM zone length nonuniformity (≤22.03 vs. >22.03)	0.035	2.03 (1.05–3.91)	0.640

BM, bone marrow; BLR, bone marrow-to-liver uptake ratio; CA19-9, carbohydrate antigen 19-9; CEA, carcinoembryonic antigen; C-index, Harrell’s concordance index; GLCM, grey-level co-occurrence matrix; GLZLM, grey-level zone length matrix; MTV, metabolic tumor volume; NLR, neutrophil-to-lymphocyte ratio; PET/CT, positron emission tomography/computed tomography; PLR, platelet-to-lymphocyte ratio; SUV, standardized uptake value; TLG, total lesion glycolysis.

**Table 3:** Multivariable analysis for predicting overall survival after adjustment for age and sex.

Variables	Model 1		Model 2	
	*p*-Value	Hazard Ratio (95% CI)	*p*-Value	Hazard Ratio (95% CI)
Clinical TNM stage				
Stage III	0.042	1.98 (1.02–4.69)	0.177	1.82 (0.76–4.36)
Stage IV	0.005	4.71 (1.59–13.95)	0.001	5.66 (2.26–15.64)
NLR	0.835	1.11 (0.41–2.98)	0.751	1.17 (0.44–3.10)
BM SUV	0.005	4.30 (1.57–11.76)	0.003	5.17 (1.76–15.16)
TLG	0.037	2.37 (1.08–5.97)	0.028	2.78 (1.12–6.95)
First-order entropy	0.013	2.89 (1.29–6.01)	-	-
GLCM energy	-	-	0.379	0.84 (0.25–1.81)
GLZLM zone length nonuniformity	-	-	0.751	1.89 (0.55–6.47)

BM, bone marrow; CI, confidence interval; GLCM, grey-level co-occurrence matrix; GLZLM, grey-level zone length matrix; NLR, neutrophil-to-lymphocyte ratio; SUV, standardized uptake value; TLG, total lesion glycolysis.

**Table 4:** Comparison of overall survival according to the PET/CT scoring system.

Score	Number of Events (%)	*p*-Value	Hazard Ratio (95% CI)	Median Overall Survival (95% CI)
Score 0–2 (*n* = 28)	8 (28.6%)	-	1.00	42.1 months (23.0–42.1 months)
Score 3 (*n* = 21)	14 (66.7%)	0.004	3.71 (1.54–8.98)	14.4 months (9.2–25.3 months)
Score 4 (*n* = 16)	15 (93.8%)	<0.001	14.52 (5.46–38.64)	7.6 months (6.9–16.8 months)

CI, confidence interval; PET/CT, positron emission tomography/computed tomography.

**Table 5:** Prognostic values of the PET/CT scoring system in subgroup analysis of overall survival.

Score		Clinical TNM Stage		Treatment	
		Stage I–II	Stage III–IV	Surgical Resection	Other Treatments
Score 0–2	*p*-value	-	-	-	-
	Hazard ratio (95% CI)	1.00	1.00	1.00	1.00
	Median overall survival (months)	42.1	34.5	42.1	34.5
Score 3	*p*-value	0.046	0.008	0.017	0.043
	Hazard ratio (95% CI)	2.96 (1.05–9.24)	6.38 (1.62–25.06)	5.03 (1.34–18.87)	3.18 (1.08–12.04)
	Median overall survival (months)	15.2	8.0	14.4	15.2
Score 4	*p*-value	0.002	<0.001	<0.001	<0.001
	Hazard ratio (95% CI)	18.54 (2.93–117.33)	12.04 (3.00–48.39)	27.40 (5.11–146.92)	11.05 (2.82–43.34)
	Median overall survival (months)	9.2	6.9	9.2	6.9

CI, confidence interval; PET/CT, positron emission tomography/computed tomography.

## Data Availability

The datasets generated during and/or analyzed during the current study are available from the corresponding authors on reasonable request.

## Supplementary Materials

The following are available online at https://www.mdpi.com/article/10.3390/cancers13143563/s1, Table S1: List of 31 higher-order textural features of primary pancreatic cancer lesion, Table S2: The results of univariable analysis for predicting overall survival in PET/CT imaging parameters of pancreatic cancer showing no statistical significance.
