# Supplementary material for: Predicting Survival in Patients with Pancreatic Cancer by Integrating Bone Marrow FDG Uptake and Radiomic Features of Primary Tumor in PET/CT
Source: Cancers (Basel). 2021 Jul 16;13(14):3563. doi: 10.3390/cancers13143563 (PMC8304187; doi:10.3390/cancers13143563)
Supplement: Supplementary file 1 [file cancers-13-03563-s001.zip › cancers-1267918-supplementary.pdf]

Table S1. List of 31 higher-order textural features of primary pancreatic cancer lesion.

Matrix	Index
Grey-level co-occurrence matrix	Contrast
	Correlation
	Dissimilarity
	Energy
	Entropy
	Homogeneity
Neighborhood grey-level different matrix	Busyness
	Coarseness
	Contrast
Grey-level run-length matrix	Grey-level non-uniformity for run
	High grey-level run emphasis
	Long-run emphasis
	Long-run high grey-level emphasis
	Long-run low grey-level emphasis
	Low grey-level run emphasis
	Run length non-uniformity
	Run percentage
	Short-run emphasis
	Short-run high grey-level emphasis
	Short-run low grey-level emphasis
Grey-level zone-length matrix	Grey-level non-uniformity for zone
	High grey-level zone emphasis
	Long-zone emphasis
	Long-zone high grey-level emphasis
	Long-zone low grey-level emphasis
	Low grey-level zone emphasis
	Short-zone emphasis
	Short-zone high grey-level emphasis
	Short-zone low grey-level emphasis
	Zone length non-uniformity
	Zone percentage

Table S2. The results of univariables analysis for predicting overall survival in PET/CT imaging parameters of pancreatic cancer showing no statistical significance.

Variables		P-value
Conventional PET/CT parameters	Maximum SUV	0.201
First-order textural parameters	Skewness	0.106
	Kurtosis	0.127
	Energy	0.087
	Sphericity	0.183
	Compacity	0.160
Grey-level co-occurrence matrix parameters	Contrast	0.226
	Correlation	0.532
	Dissimilarity	0.295
	Homogeneity	0.499
Neighborhood grey-level different matrix parameters	Busyness	0.749
	Coarseness	0.070
	Contrast	0.063
Grey-level run-length matrix parameters	Grey-level non-uniformity for run	0.240

	High grey-level run emphasis	0.078
	Long-run emphasis	0.221
	Long-run high grey-level emphasis	0.403
	Long-run low grey-level emphasis	0.380
	Low grey-level run emphasis	0.792
	Run length non-uniformity	0.454
	Run percentage	0.271
	Short-run emphasis	0.165
	Short-run high grey-level emphasis	0.078
	Short-run low grey-level emphasis	0.775
Grey-level zone-length matrix parameters	Grey-level non-uniformity for zone	0.308
	High grey-level zone emphasis	0.164
	Long-zone emphasis	0.197
	Long-zone high grey-level emphasis	0.295
	Long-zone low grey-level emphasis	0.184
	Low grey-level zone emphasis	0.902
	Short-zone emphasis	0.124
	Short-zone high grey-level emphasis	0.237
	Short-zone low grey-level emphasis	0.062
	Zone percentage	0.114

PET/CT, positron emission tomography/computed tomography; SUV, standardized uptake value
